# Experimental study on ultrasonic irradiation for enhancing coalbed methane recovery

**DOI:** 10.1038/s41598-022-11948-5

**Published:** 2022-05-11

**Authors:** Xin Ding, Jing Hou, Xiaochun Xiao

**Affiliations:** 1 School of Mechanics and Engineering, Liaoning Technical University, Fuxin, 123000 China; 2 Liaoning Key Laboratory of Mining Environment and Disaster Mechanics, Liaoning Technical University, Fuxin, 123000 China

**Keywords:** Fossil fuels, Fuel cells

## Abstract

The present study proposes the use of a new ultrasonic irradiation method to enhance permeability and desorption for gas recovery from low-permeability coal reservoirs. A triaxial stress ultrasonic irradiation test apparatus was developed specifically for coal, considering the properties of gas adsorption, migration, and sound intensity, and providing a simultaneous measurement of gas flux, to investigated the deformation and temperature of coal samples obtained from the Fuxin coal field by permeability and desorption experiments. With the ultrasonic irradiation duration, the permeability of coal improved gradually with unequal variation, accompanied by the Klinkenberg effect where it decreased rapidly and then increased slowly with increasing gas pressure. The ability to desorb coal was enhanced by higher sound intensity ultrasound irradiation, and the volume of gas desorption was much greater than that of the sample without mange, the temperature and strain were demonstrated as a “J shaped” curve. An X-ray computer tomography (CT) technique was used to visualise the meso- or macro-cracks in the coal sample at pre- and post- ultrasonic irradiation, consequently, fractures expanded under the irradiation of ultrasonic waves. A permeability and desorption model was developed to describe the improvement of coal seam gas production capacity under ultrasonic irradiation, which introduced effective sound pressure.

## Introduction

Natural gas accounts for more than 20% of the world’s energy consumption, and coalbed methane is an important component in many countries, such as the USA, Canada, Australia, and China. With rapid economic development in the future, the demand for energy is increasing daily, which requires new mining technologies to ensure the stable production of coalbed methane.

Gas desorption caused by changes in ambient gas pressure leads to the breaking of adsorption dynamic equilibrium and seepage produced by the gas gradient, which are the two key processes of gas production affected by the void in coal. The space in coal seams results from coal metamorphism and geostress evolution, limiting the recovery of methane through both downhole drilling in the working face of deep mines and surface gas well drainage. The phenomena of gas adsorption–desorption and matrix swelling–shrinkage caused the gas migration in coal to be much more complex than other rock types. A number of studies have previously focused on the permeability evolution influence by water, stress, gas pressure, and other conditions through physical experimentation and theoretical analysis, which is the key parameter of methane well deliverability in low-permeability coal reservoirs^1–14^. They found that gas migration in coal seams is accompanied by the behaviours of mechanics and non-mechanics, and there is a larger flow space, i.e., higher permeability can guarantee gas desorption and better continuous directional movement, with the inhibition of stress and coal expansion, as well as the enhancement of matrix shrinkage. Thus, improving the gas migration characteristics of coal reservoirs is still a key issue in energy engineering, for both coalbed methane development and gas outburst hazard prevention in deep mining. In order to improve the coal seam permeability and obtain a long-term and stable gas source, many scholars have performed research on enhanced gas permeability in both the field and laboratory, through processes such as waterjet, enhanced microbial, CO2 injection, microwave irradiation, and liquid nitrogen cryogenic freeze^15–29^. This new method improved the seepage capacity of coal, but its applicability and effectiveness require further study.

Ultrasound is a sound wave with a frequency higher than 20,000 Hz and is widely used in medicine, military, industry, and agriculture because of its good directionality, strong penetration ability, and easily obtained concentrated sound energy^30^. Two phenomena occur in the material: the mechanical effect caused by a high number of cycles, leading to tensile and compressive stress; and a thermal effect that causes the temperature to rise results in non-uniform deformation during ultrasonic irradiation, which promotes crack propagation in rocks and increases fluid migration space^31^, accelerating gas desorption on the surface of the coal matrix^32,33^. Therefore, ultrasonic irradiation can be used as a new method to improve the gas permeability of coal and promote the gas seepage capacity, for underground gas in-situ extraction after roadway excavation.

In the present study, further investigations into the permeability of coal samples with ultrasonic irradiation and gas flux tests were conducted using a triaxial stress test apparatus at different confining and gas pressures. The simultaneous strain and temperature were also measured. The internal fractures in coal before and after ultrasonic irradiation were observed by computer tomography (CT). Mechanical models were then developed to describe the permeability and desorption of reservoirs under the influence of the sound intensity. Based on this, coal source exploitation can eliminate gas anomaly accumulation and improve the efficiency of coalbed methane reservoirs, which reduces greenhouse gas emissions and contributes to more natural gas production.

## Experiments

### Sample preparation

**Figure 1:**
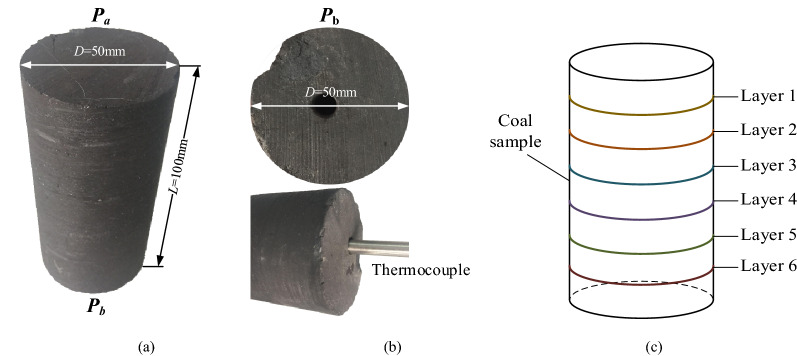
Coal materials. (**a**) Coal sample, (**b**) end of sample with a hole, (**c**) Layer positions of CT slice images.

The bottle coal bulks were collected from the Wulong Colliery to a depth of 950 m and immediately sealed with valve bags. The study area is located in the middle of Fuxin Coalfield, Liaoning Province, China, which is one of the most successful areas for the commercial development of coalbed methane in China. The coal reservoir gas content was 11,167 m^3^/t, with an average porosity of 4.7% and average density of 1189 kg/m^3^. Cylindrical specimens measuring *ϕ* 50 mm × 100 mm were prepared in the laboratory, in which the coring direction was perpendicular to that of bedding development, and to weaken the influence induced by the difference between the two ends, both ends of each specimen were polished to ensure that the flatness error was less than ± 0.02 mm, using a drill bit to obtain a hole with 8 mm diameter and 50 mm depth at the centre of the sample end to place the thermocouple, as shown in Fig. 1a and b. Table 1 summarises the physicochemical composition of the coal. X-ray diffraction (XRD) showed that more minerals were observed, mainly quartz and clay, pyrite, and calcite, as secondary compositions in the coal. The aim of this study was to investigate whether ultrasonic stimulation increases permeability; therefore, the effect of clay swelling on the strain of coal under water-saturated conditions is negligible.

**Table 1:** Properties and proximate elemental analysis of tested coal.

Fixed carbon (*FC*_ad_)	Volatile matter (*V*_ad_)	Ash content (*A*_ad_)	Moisture content (*M*_ad_)
**Proximate analysis**			
65.91%	21.06%	8.25%	4.78%

Carbon (C)	Hydrogen (H)	Nitrogen (N)	Sulfur (S)
**Ultimate analysis**			
83.11%	5.23%	1.27%	0.7%

Vitrinite	Silk carbonized material		Keratinates
**Maceral composition**			
73.12%	15.84%		1.37%

Quartz	Clay	Pyritic	Calcite
**Constituent of mineral matter**			
3.64%	2.58%	1.19%	0.87%

Superscripts: “ad” means results were obtained by air dry coal sample (*FC*_ad_, *V*_ad_, *A*_ad_, *M*_ad_).

### Experimental setup

**Figure 2:**
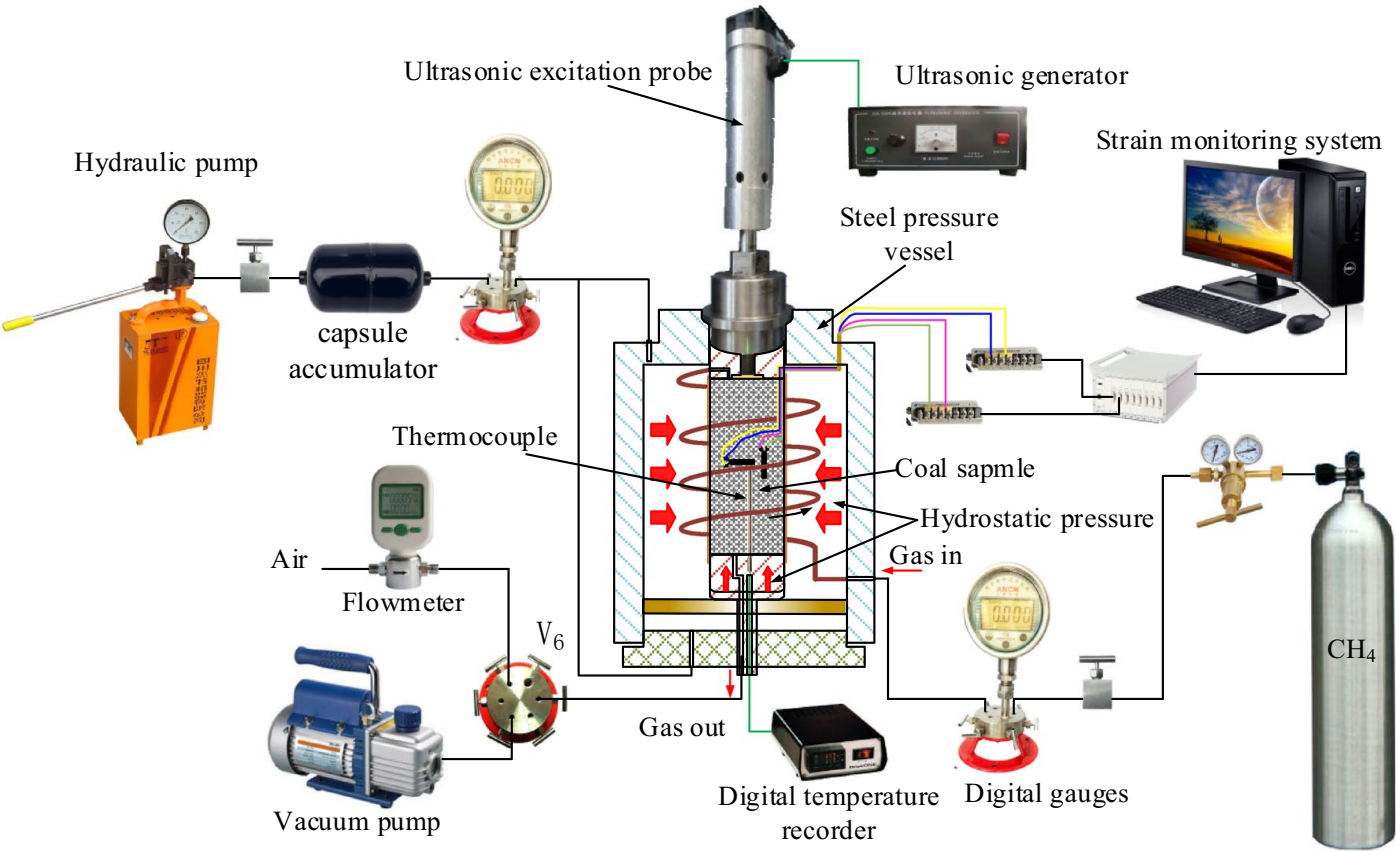
Schematic diagram of the flow velocity–pressure of coal under ultrasonic irradiation experimental setup.

A triaxial ultrasonic irradiating test apparatus was developed specifically for stressed coal, taking the properties of gas adsorption, migration, and sound intensity into consideration under stress conditions and providing a simultaneous measurement of gas flux. The test system includes five parts, as shown in Fig. 2:

1. A pressure chamber applies hydrostatic pressure to the coal sample

Steel pressure vessel was cut from manganese steel and dimensions was height 300 mm, outer diameter 200 mm, inner diameter 150 mm, with a safety design pressure resistance of 50 MPa. A hydrostatic loading apparatus was used to pump in fluid medium to the axial and circumferential directions to bring the coal sample to hydrostatic pressure and the power source was a hydraulic pump by using water, which has a pressure transmission capacity of 40 MPa, digital gauges recorded the pressure (full scale 25 MPa, with a resolution of the number of impressions at 5‰), and a capsule accumulator stabilised the pressure in the system. The device has good sealing and experimental accuracy in the temperature range of 10–50 °C.

(2) A gas monitoring system applied gas adsorption and directional flow while recording gas migration.

As nitrogen and methane have similar properties in coal adsorption and migration^34^, a stable pure nitrogen gas source with purities of 99.999% is used for seepage and adsorption in the coal for safety during testing. An air regulator, a high-accuracy digital gauge (full scale 25 MPa, with a precision of 0.05%), and an air valve are connected sequentially by a stainless-steel pipeline, then to a gas channel on the pressure cell. Gas seepage through the coal occurs via a stainless-steel pipeline and enters a six-way valve, which is connected to a gas flowmeter to monitor the gas pressure, and the flux data are recorded per second and resolution is 0.001 m/s. The other method includes a connection to a vacuum pump to remove gas impurities from the coal samples before the trials.

(3) A strain monitoring system detected and recorded axial and circumferential strains.

The axial and circumferential deformations of the sample were measured by two BX120-15AA resistance strain gauges that were perpendicular to each other and attached to the surface of the sample, which test accuracy of strain is 10^−6^, the digital signals of the axial and circumferential strains were collected using a DHC 5995 h dynamic strain indicator for real-time observations, manufactured by Donghua Technology, Ltd., display, and recorded by a high-speed computer. The average tolerance of the strain gauge was 0.1%, and the sensitivity coefficient was 1%. The sampling frequency was set to 0.1 Hz.

(4) A temperature monitoring system recorded the temperature variation of the coal sample.

A thermocouple was fixed on the lower gripper, the heat sensitive part probed the hole in the sample (measuring range − 20 to 130 °C, thermal response time is 0.01 s, and error is 0.005 °C), as shown in Fig. 1b, and the wire external temperature display module recorded every 30 s.

(5) An ultrasonic generating system applied ultrasonic irradiation with different sound intensities to the sample.

The ultrasonic generating system contained an ultrasonic generator and an ultrasonic irradiation probe. The ultrasonic irradiation probe was fixed on the upper gripper, and a high-strength steel disc was placed between it and the specimen to prevent local stress concentration on the surface of the sample. The ultrasonic generator provides three sound intensities to the probe, according to Eq. (1) and listed in Table 2, which can be shifted by a gear as needed. The system has a fixed ultrasonic frequency, in the paper is 30 kHz, and the sound intensity of ultrasonic does not change with frequency, so it was not included in present study. In addition, the sound intensities were determined according to the basic performance parameters, the equipment we used had 3 current gears for selection.

1 $$ J_{0} = \frac{UIt}{{St}} = \frac{UI}{S} $$

**Table 2:** Parameters of sound intensity.

Gear position	Current *I* (A)	Voltage *U* (V)	Probe area *S* (cm^2^)	Sound intensity *J*_0_ (W cm^−2^)
1	0.75	110	1.99	41.45
2	1.05	110	1.99	58.04
3	1.10	110	1.99	60.80

### Experimental program and procedure

The experiment involved gas adsorption and flux testing of coal samples at different confining and gas pressures, ultrasonic sound intensities, and irradiation durations; the confining and gas pressures and the sound intensity gear positions are shown in Table 3. The experiment in the present study consists of two parts: one constitutes permeability measurement, as shown in Table 3 test types I and II, the other constitutes desorption measurement as shown in Table 3 test type III. The following procedure was conducted:

1. Sample pretreatment and installation: Strain gauges of electrical resistance were mutually perpendicular to each other and placed on the surface of the coal. The thermocouple was fixed on the lower sample holder and in close contact with the upper and lower holder with a thermoplastic sleeve covered on the surface to separate the confining pressure from the pore and placed in the pressure cell. The ultrasonic irradiation probe was placed on the top of the steel pressure vessel and connected to all instruments and sensors.
2. Pressure was applied, and gas adsorption occurred. First, the steel pressure vessel was filled with water with alternating axial loads equal to the confining pressures of the hydrostatic pressure state as predetermined (*σ*_1_ = *σ*_3_ = 4, 8, and 12 MPa). After closing the inlet switch and opening the six-way valve connected to the vacuum pump, the coal sample was placed under a vacuum for 8 h to extract the impure gases. Subsequently, the inlet switch was opened, the six-way valve was closed, and the gas flowed into the coal sample to a predetermined pressure (*P*_a_ = 1.0 MPa) and was adsorbed for 24 h to saturation.
3. Percolation: While maintaining the hydrostatic pressure, the irradiating gear of the ultrasonic generator was increased to the predetermined values, simultaneously recording the deformation of coal and the temperature. After different ultrasonic irradiation durations (*t* = 2, 4, and 12), the inlet opening switch and the six-way valve were turned to the flowmeter, the gas pressure (*P*_a_ = 0.2, 0.5, 0.8, 1.0, 1.2, and 1.5 MPa) was gradually adjusted, and the gas pressure and flux data were recorded.
4. Desorption: The sample was kept under hydrostatic pressure, the inlet switch was closed, and the six-way valve was opened to the air until the free gas in the coal was discharged and then entered the flowmeter. Simultaneously, the irradiating gear of the ultrasonic generator increased to the predetermined values, or no irradiation, recording the deformation of coal and the temperature, and obtaining gas desorption by drainage gas collection.
5. The tests were repeated by changing the confining pressure, gas pressure, and sound intensity according to the testing scheme. Procedures (1), (2), and (3) were repeated after replacing the coal sample for permeability measurement, and procedures (1), (2), and (4) were repeated after replacing the coal sample for desorption measurement.

**Table 3:** Test scheme.

Type	Coals	Hydrostatic pressure *σ*_1_ = *σ*_3_ (MPa)	Gas pressure *P*_a_ (MPa)	Gear position	Duration *t* (h)
I	^#^1	4	0.2, 0.5, 0.8, 1.0, 1.2, 1.5	1	2, 4, 12
	^#^2	8			
	^#^3	12			
II	^#^4	4	0.2, 0.5, 0.8, 1.0, 1.2, 1.5	1	2, 4, 12
	^#^5			2	
	^#^6			3	
III	^#^7	4	1.0	No	–
	^#^8			1	
	^#^9			2	
	^#^10			3	

Superscripts: the ultrasonic irradiating is always performed throughout the desorption process.

It is worth noting that all experiments were conducted in the laboratory at room temperature (20 °C), which was maintained at a constant by air conditioning. In addition, the internal pore structure of the coal before and after ultrasonic irradiation was observed using CT. We assume that gas permeation through specimens is an isothermal process, and that coal can be regarded as an isotropic homogeneous material. Therefore, the gas permeability can be expressed by Darcy’s law:

2 $$ k = \frac{2\mu LQ}{{A(P_{a}^{2} - P_{b}^{2} )}} $$

where *k* is the permeability (mD); *Q* is the velocity of the gas flow (cm^3^/s); *A* is the average cross-sectional area of the specimen (cm^2^); *L* is the length of the specimen (mm); *P*a is the inlet gas pressure (MPa); *P*b is the outlet gas pressure (MPa); and *μ* is the gas viscosity (Pa s, *μ* (N^2^) = 10.093 Pa s).

## Experimental result

### Resultant permeability, strain, and temperature at different hydrostatic pressures

**Figure 3:**
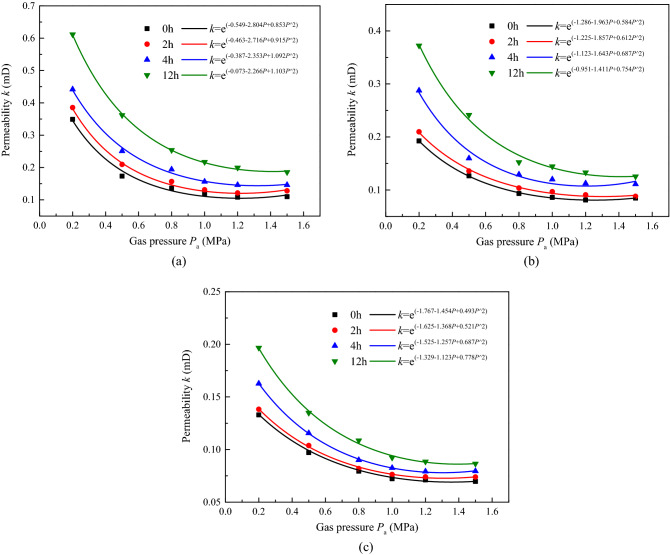
Permeability of coal with different hydrostatic pressure and irradiating. (**a**) *σ*_1_ = *σ*_3_ = 4 MPa, (**b**) *σ*_1_ = *σ*_3_ = 8 MPa, (**c**) *σ*_1_ = *σ*_3_ = 12 MPa.

**Figure 4:**
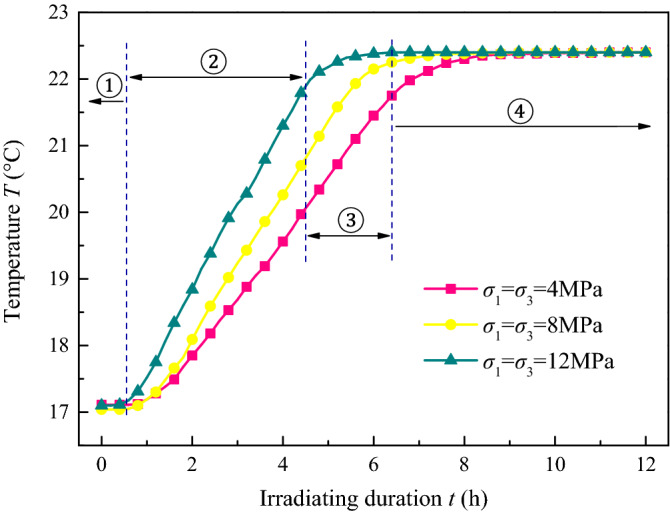
Temperature of coal with time under ultrasonic irradiating with different hydrostatic pressure.

**Figure 5:**
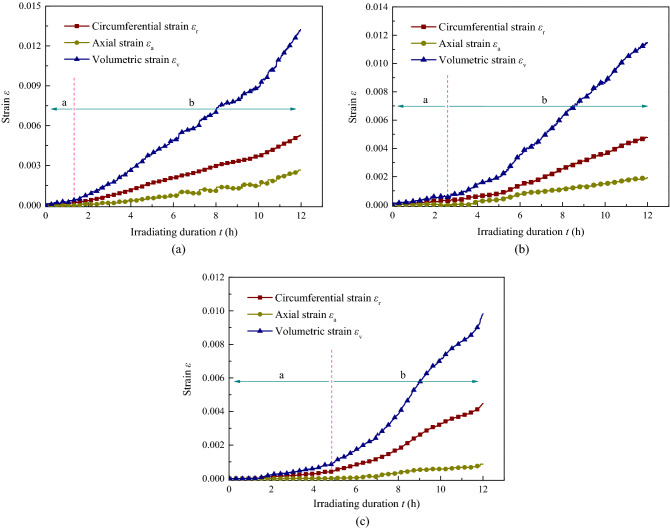
Strain of coal with time under ultrasonic irradiating with different hydrostatic pressure. (**a**) *σ*_1_ = *σ*_3_ = 4 MPa, (**b**) *σ*_1_ = *σ*_3_ = 8 MPa, (**c**) *σ*_1_ = *σ*_3_ = 12 MPa.

According to plan type I presented in Table 3, Fig. 3 shows the permeability of the coal sample with low hydrostatic pressure (sample ^#^1, *σ*_1_ = *σ*_3_ = 4 MPa), medium pressure (sample ^#^2, *σ*_1_ = *σ*_3_ = 8 MPa), and high pressure (sample ^#^3, *σ*_1_ = *σ*_3_ = 12 MPa) under different irradiation durations (*t* = 0, 2, 4, and 12 h); Figs. 4 and 5 show the temperature (*T*) and strain (*ε*) variation with time, and Table 4 shows the corresponding growth rate of permeability, where the ultrasonic irradiation sound intensity is *J*_0_ = 3.05 W cm^−2^.

**Table 4:** Growth rate of permeability of coal under ultrasonic irradiating with different hydrostatic pressure.

Hydrostatic pressure *σ*_1_ = *σ*_3_ (MPa)	Gas pressure *P*_a_ (MPa)	No	Irradiating duration 2 h		Irradiating duration 4 h		Irradiating duration 12 h	
		Permeability *k* (mD)	Permeability *k* (mD)	Growth rate (%)	Permeability *k* (mD)	Growth rate (%)	Permeability *k* (mD)	Growth rate (%)
4	0.2	0.349	0.382	9.45	0.438	25.50	0.610	74.78
	0.5	0.174	0.213	22.41	0.251	44.25	0.366	110.34
	0.8	0.136	0.157	15.44	0.194	42.64	0.253	86.03
	1.0	0.119	0.126	5.88	0.157	31.93	0.215	80.67
	1.2	0.108	0.120	11.11	0.146	35.18	0.198	83.33
	1.5	0.110	0.131	19.09	0.146	32.72	0.189	71.81
8	0.2	0.192	0.208	8.33	0.287	49.48	0.373	94.27
	0.5	0.126	0.139	10.31	0.159	26.19	0.242	92.06
	0.8	0.093	0.105	12.90	0.129	38.71	0.152	63.44
	1.0	0.086	0.093	8.14	0.120	39.53	0.141	63.95
	1.2	0.081	0.088	8.64	0.112	38.27	0.133	64.19
	1.5	0.084	0.090	7.14	0.111	32.14	0.126	50.00
12	0.2	0.132	0.138	4.54	0.163	23.48	0.196	48.48
	0.5	0.097	0.104	7.21	0.116	19.58	0.135	39.17
	0.8	0.079	0.082	3.79	0.090	13.92	0.108	36.71
	1.0	0.072	0.076	5.55	0.082	13.89	0.092	27.77
	1.2	0.071	0.074	4.22	0.079	11.26	0.088	23.94
	1.5	0.069	0.073	5.79	0.080	15.94	0.086	24.63

As shown in Fig. 3, there are similar trends of permeability for coal samples under different hydrostatic pressures; permeability decreases rapidly at first and then increases slightly, displaying “V shaped” variation characteristics with increasing gas pressure. The main reason for this phenomenon is the Klinkenberg effect in coal, under low gas pressure, the coal acts as a type of porous medium, and there is no significant difference in the velocity of gas molecules between the centre of the channel and the wall, furthermore, the mean free path of the gas molecules reaches the sides of the channel, and the diffusion of gas molecules freely occurs without collision, resulting in considerable initial permeability. However, this effect faded as the gas pressure increased, and the permeability gradually declined, even under ultrasonic irradiation. As the ultrasonic irradiation duration increased, the permeability of coal gradually improved at each gas pressure with unequal variation. By conducting quotients on the change and initial permeability, the quantitative results of the permeability growth rate were obtained and are listed in Table 4. This clearly indicates that the growth rate increases with increasing irradiation duration; for example, the growth rates of sample ^#^1 were 22.4%, 44.25%, and 110.34% after 4, 8, and 12 h of irradiation, respectively. However, it also demonstrated that the growth rate of permeability decreases with improving gas pressure and higher hydrostatic pressure; for example, the growth rates of sample ^#^3 were 23.48%, 19.58%, 13.92%, 13.89%, 11.26%, and 15.94% after 12 h of irradiation with increasing gas pressure, respectively. For samples ^#^1, ^#^2, and ^#^3, the growth rates were 71.8–110.34%, 50.00–94.27%, and 23.94–48.48% after 12 h of irradiation with increasing gas pressure, respectively. It means that, ultrasonic irradiation did improved the permeability of coal, for the mechanical effect periodic of loading and unloading during ultrasonic transmission, cracks were formed between coal particles, which has been confirmed by research^35^, provided much new effective channels for gas migration and the longer irradiating duration, the better cracks formation in coal. However, the promotion slows down with the inhibition of external confining pressure, for the increase of critical crack growth stress. In general, the impact of a longer irradiation duration is quite beneficial for increasing the permeability of the coal reservoir, however, it generated much small fractures and cannot change the fundamental seepage law of the reservoir, which is related to the Klinkenberg effect, and the higher hydrostatic pressure inhibiting it. In this part, the variation of permeability with gas pressure under ultrasonic irradiation can be expressed as:

3 $$ k = e^{{(a + bP + cP^{2} )}} $$

where *a*, *b*, *c*, are the fitting parameters (mD, mD/MPa, mD/MPa^2^,) and related to the ultrasonic duration.

The temperature was recorded by the thermocouple and is expressed as an “S shaped” curve and dived into 4 stages, as demonstrated in Fig. 4: (1) initial temperature stabilisation, (2) stable warming, (3) slow warming, and (4) constant temperature phase during the 12 h of irradiation. In the initial temperature stabilisation phase (1), the curve was approximately a straight line where the temperature recorded only a slight rise at 17.1–17.3 °C. In the stable warming phase (2), the temperature increased with a relatively stable growth rate. The slow warming phase (3), wherein the curve improved with a gradually diminishing growth rate, trended to 0, and was concave downward. Finally, in the constant temperature phase (4), the temperature, basically stable at 22.4 °C, was an approximately horizontal line. The effect of the hydrostatic stress state was mainly reflected in the stable warming and the slow warming stages, unexpectedly, when the sample was under a higher stress state, the shorter the warming duration lasted, the faster the temperature rose at the stable warming phase and the constant temperature phase. The results for samples ^#^1, ^#^2, and ^#^3 were approximately 0.67 °C/h, 0.89 °C/h, and 1.25 °C/h, respectively. There are two effects in coal with ultrasonic irradiating, which the fatigue effect and thermal effect, former is dissipated as crushing energy to promotes cracks development, and the latter is converted into heat to increase the temperature of the coal. When the input energy caused by ultrasonic irradiating was constant in unit time, according to the previous results that the permeability increment was small at high stress, it means more energy was converted into heat and less to dissipation as cracks development. As a result, the samples with a higher stress state shown a shorter warming duration lasted and faster the temperature rose.

The axial, circumferential, and volumetric strains are shown in Fig. 5, and all the values were positive, which implies that the entire behaviour of coal under irradiation exhibited swelling deformation. A similar variation was seen based on the volumetric strain, in general. The micro-expansion phase (a) of the samples has a smaller value and growth rate in the first few hours, and the duration is gradually prolonged by the effect of the hydrostatic pressure increase. The measurements of samples ^#^1, ^#^2, and ^#^3 were 1.4, 2.5, and 4.8 h, respectively. Thereafter, the slope of the strain curve increased, and the total strain gradually recovered with little fluctuation to enter the stable macro-expansion stage (b) represented by the dotted red line, where a smaller strain growth rate takes shape with a larger hydrostatic pressure. As a result, the volumetric strain of sample ^#^3 was 0.00982, and that of sample ^#^1 was 0.01324. In addition, it is clearly seen that the axial strain was always shorter than that formed by the circumferential strain in all experiments, and the latter entered the stable macro-expansion stage before the former. On the one hand, for the reasons of this phenomenon, numbers of cracks emerged and opened in coal for the fatigue effect of ultrasonic irradiating, and then expansion deformation was caused, the relationship between cracks and deformation was confirmed by the article^8^. On the other hand, it was easy to found that, the deformation formed lag behind the change of temperature, compared with the results in the previous, with the increased of temperature, the thermal expansion of a large number of matrix particles forms macro deformation was monitored. However, the higher the pressure, the greater the inhibition of deformation, as well as the coal at low confining pressure, the faster the deformation speed, the greater the deformation.

**Figure 6:**
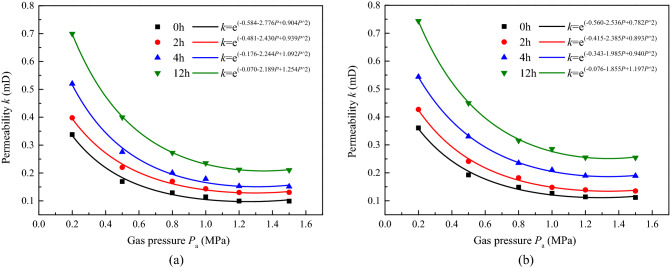
Permeability of coal with time under different ultrasonic irradiating sound intensity. (**a**) *J*_0_ = 58.04 W cm^−2^, (**b**) *J*_0_ = 60.80 W cm^−2^.

**Figure 7:**
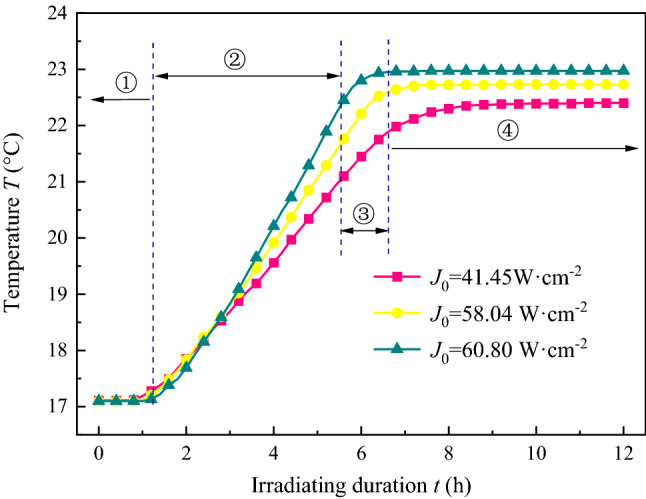
Temperature of coal with time under ultrasonic irradiating with different hydrostatic pressure.

Figures 6 and 7, in combination with Figs. 4a and 5a, demonstrate that when the permeability and strain of samples were under a hydrostatic pressure of 4 MPa at different ultrasonic irradiation gear positions, the sound intensities were 41.45, 58.04 and 60.80 W cm^−2^ for samples ^#^1, ^#^5 and ^#^6, respectively.

The evolution of permeability with gas pressure still conformed to the seepage law influenced by the Klinkenberg effect, except that the spacing between curves of different irradiation durations increased with a higher sound intensity and enabled coal to pass gas more easily. As shown in Table 5, the growth rate increased with higher sound intensity, with that of samples ^#^1, ^#^4, and ^#^6 being 71.81–110.34%, 106–136.09%, and 105.81–133.85%, respectively, after 12 h of irradiation.

**Table 5:** Growth rate of permeability of coal under different ultrasonic irradiating sound intensity with hydrostatic pressure *σ*_1_ = *σ*_3_ 4 MPa.

Sound intensity *J*_0_ (W cm^−2^)	Gas pressure *P*_a_ (MPa)	No	Irradiating duration 2 h		Irradiating duration 4 h		Irradiating duration 12 h	
		Permeability *k* (mD)	Permeability *k* (mD)	Growth rate (%)	Permeability *k* (mD)	Growth rate (%)	Permeability *k* (mD)	Growth rate (%)
41.45	0.2	0.349	0.382	9.45	0.438	25.50	0.610	74.78
	0.5	0.174	0.213	22.41	0.251	44.25	0.366	110.34
	0.8	0.136	0.157	15.44	0.194	42.64	0.253	86.03
	1.0	0.119	0.126	5.88	0.157	31.93	0.215	80.67
	1.2	0.108	0.120	11.11	0.146	35.18	0.198	83.33
	1.5	0.110	0.131	19.09	0.146	32.72	0.189	71.81
58.04	0.2	0.337	0.398	18.10	0.520	54.30	0.699	107.41
	0.5	0.169	0.220	30.17	0.275	62.72	0.399	136.09
	0.8	0.129	0.169	31.00	0.200	55.03	0.272	110.85
	1.0	0.114	0.143	25.43	0.178	56.14	0.235	106.14
	1.2	0.099	0.130	31.31	0.152	53.53	0.212	114.14
	1.5	0.099	0.129	30.30	0.151	52.52	0.210	112.12
60.80	0.2	0.361	0.426	18.01	0.543	50.41	0.743	105.81
	0.5	0.192	0.241	25.52	0.330	71.87	0.449	133.85
	0.8	0.148	0.182	22.97	0.235	58.78	0.315	112.84
	1.0	0.126	0.148	17.46	0.210	66.66	0.285	126.19
	1.2	0.114	0.139	21.93	0.189	65.79	0.255	123.68
	1.5	0.112	0.135	20.53	0.188	67.85	0.254	126.78

As presented in Fig. 7, the performance curve was “S shaped”, with 4 stages. For the irradiation performed with an increasing sound intensity, the sample curves were observed with a larger growth rate in the stable warming process, shorter slow warming phase, and higher temperature continued. The observed temperatures for samples ^#^1, ^#^5, and ^#^6 were 22.97 °C, 22.73 °C, and 22.35 °C, respectively. Thus, we can see that under the irradiation at higher sound intensity, it is easier for the sample to reach a higher equilibrium temperature in a short time.

**Figure 8:**
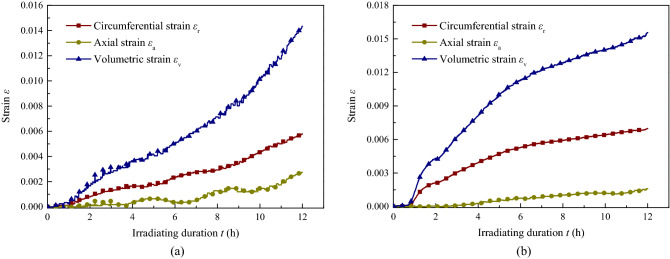
Strain of coal with time under triaxial stress with different sound intensity ultrasonic irradiating. (**a**) *J*_0_ = 58.04 W cm^−2^, (**b**) *J*_0_ = 60.80 W cm^−2^.

As shown in Fig. 8, the strain of the samples can be divided into 2 phases: micro-expansion and stable macro-expansion. The former was gradually shortened as the sound intensity improved, and the growth rate of the latter increased. For samples ^#^4 and ^#^5, the first phase lasted 1.3 h and 0.8 h, respectively. In addition, the sample with a higher sound intensity showed a larger strain growth rate and developed more overall strain, especially in the circumferential direction.

It can be found that, for the two effects of ultrasonic irradiation improved as the effective sound pressure increased, where the permeability, temperature and strain turn faster and larger, for there was much more ultrasonic energy is converted into heat through the friction between the particles, the interface, and the absorption of the medium. And the grater mechanical vibration accompanied by the relative displacement of the matrix and mutual friction. It is worth noting that the mechanical effect is the main mechanism of material fracture, by which tensile stress is generated by transverse waves and shear stress is generated by longitudinal waves between the matrix, where the cracks of types I and II are initiated and developed^36^. In the present experiments, when the effective sound pressure was constant, at a certain value of the total energy input, for samples with a higher pressure state, a larger fracture toughness, and increased critical expansion stress, few cracks developed, surface energy dissipated, and most of the energy was converted to heat, resulting in a larger rate of temperature increase and lower recorded strain; when the heat transfer from the coal to the outside was the same as the energy generated by the ultrasonic wave, the temperature tended to be a fixed value. Conversely, a higher sound intensity irradiation had more energy input into the coal, and a larger stress formed between the matrix; thus, a larger growth rate and higher equilibrium of temperature and strain were recorded.

### Desorption volume, strain, and temperature of different irradiation durations

**Figure 9:**
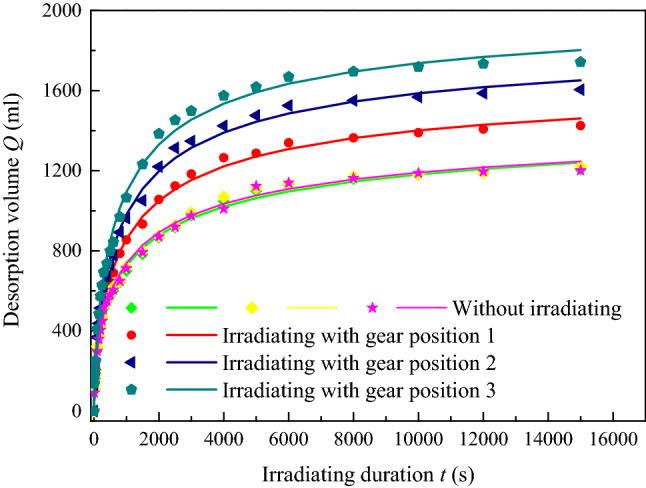
Desorption volume of coal with time under with different sound intensity ultrasonic irradiating.

**Figure 10:**
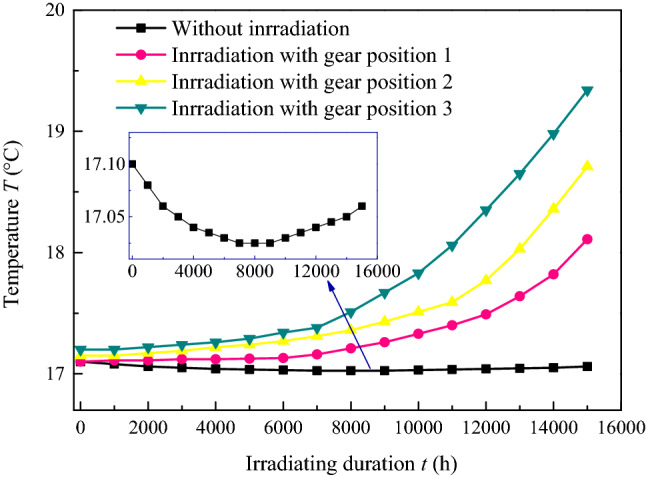
Temperature of coal with time under ultrasonic irradiating with different hydrostatic pressure during gas desorption.

**Figure 11:**
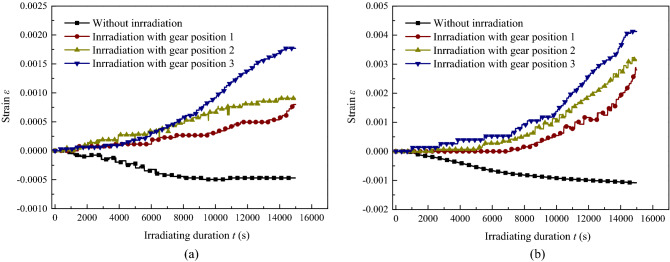
Strain of coal with time during gas desorption. (**a**) Axial strain, (**b**) Circumferential strain.

The results of the gas desorption volume, temperature, and strain of the coal samples are shown in Figs. 9, 10, and 11 for the experiments conducted at an adsorption gas pressure of 1.0 MPa and hydrostatic pressure of 4.0 MPa with or without irradiation at different sound intensities.

As shown in Fig. 9, the gas desorption volume increased rapidly at first, and as time progressed, the slope of the curves decreased gradually, resulting in a lower desorption rate. The total desorption volume decreased to an approximate stable value, and the major desorption of the samples used in the present study was concentrated in the first 8000 s, more than 95% of the total. It can be seen that the desorption volume of the irradiated sample was greater than that of the sample without irradiation, and it increased with higher sound intensity. This demonstrates that the ability to gas desorb from coal is enhanced by ultrasound irradiation. More adsorbed gas molecules deviate from the surface of the coal matrix and move to the lower gas pressure in the free state, through the pores and fissures inside the coal, displaying a higher rate of desorption and greater total volume for the macro phase.

Figure 10 shows the temperature of the samples with and without irradiation. It is noteworthy that, the temperature of the sample without irradiation recorded during the gas desorption is shown as a “V shape” that decreases after the subsequent increase in the range of 0.1 °C, and the inflexion point is approximately 7000–9000 s. The other results were “J shaped” curves, in which the growth gradient increased gradually, and the temperature gradient and the final were improved with a larger sound intensity. The process of gas sorption and desorption from the coal matrix is accompanied by temperature fluctuations, which has been confirmed in many studies^37,38^ and is caused by the release and absorption of energy. More energy was transformed from coal into gas due to its initial free energy with a larger desorption rate, then, the recorded temperature decreased and was lower than normal. As resolution gradually decreased, less energy was needed, the outsider energy transformed into samples, and the temperature of the sample rose and drew towards normal. In contrast, the ultrasonic thermal effect provides more energy for the adsorbed gas molecules to move freely and desorb from the coal matrix, more free gas transport into the outer free space. Moreover, ultrasonic irradiation causes significant thermal effects in coal and increases with longer duration and higher sound intensity, and the recorded temperature curve in coal has smaller growth gradients in the early stages and higher growth gradients in the later stages, when the gas desorption endotherm is considered. Furthermore, there was much small cracks emerged, result in the fatigue effect caused by a high number of periodic irradiation, which provided storage space and migration channel for gas desorption to free, and it had more gas desorption amount per unit time and total amount.

The strain of the samples can be seen in Fig. 11, which shows a similar pattern of axial and circumferential strain variation; the strain variation of sample ^#^7 without irradiation had an obvious negative value and shrinkage with gas desorption from the coal, which showed a larger shrinkage rate in the first 6000–8000 s and gradually slowed down in the latter stage. In contrast, the strains of the samples with irradiation all were positive and expanded over the entire process, where a smaller growth rate initially and slowly increased, and then, the strain presented a steady expansion after 6000 s. The strain of the samples increased with sound intensity, both axially and circumferentially, and the circumferential strain was much greater than the axial strain, as that shown before on gas seepage. With ultrasonic irradiation, in general, the strain of coal with gas desorption is lower than that of samples with gas-saturated adsorption.

Due to the occurrence of the phenomenon during gas desorption in this part, the matrix shrinkage of coal is accompanied by gas desorbed from the surface of the coal matrix^39^, especially at the initial rapid desorption stage, where the negative strain of shrinkage characteristics came into being. A small decrease in temperature with gas desorption was recorded at present, which produced more shrinkage. On the one hand, the thermal effect gradually increased with the positive strain during the ultrasonic irradiation of the sample, as we concluded before in this paper, and it also inhibited gas desorption, while on the other, the fracture was caused by mechanical effects, in which more strain and cracks were created for the free gas swarm and migration, promoting the dynamic balance of desorption to the positive development. During the gas desorption of coal with ultrasonic irradiation, coupling not only shrinks but also swells. The strain is the comprehensive result of the shrinkage and swelling effect in the macro scale. As a result, in the first period with a high desorption rate, the axial and circumferential strain experienced little increase, and it will take longer with a lower sound intensity irradiation; in the second period with a low desorption rate, the strain variation increased as the growth rate continued to rise. For a larger temperature growth rate caused by a higher sound intensity, it is easier to reach a higher temperature of the sample with shorter irradiation duration, larger strain emerged at the same time, and eventually more gas was collected.

## Discussion of the mechanism promoting the permeability and desorption enhancement by ultrasonic irradiation

**Figure 12:**
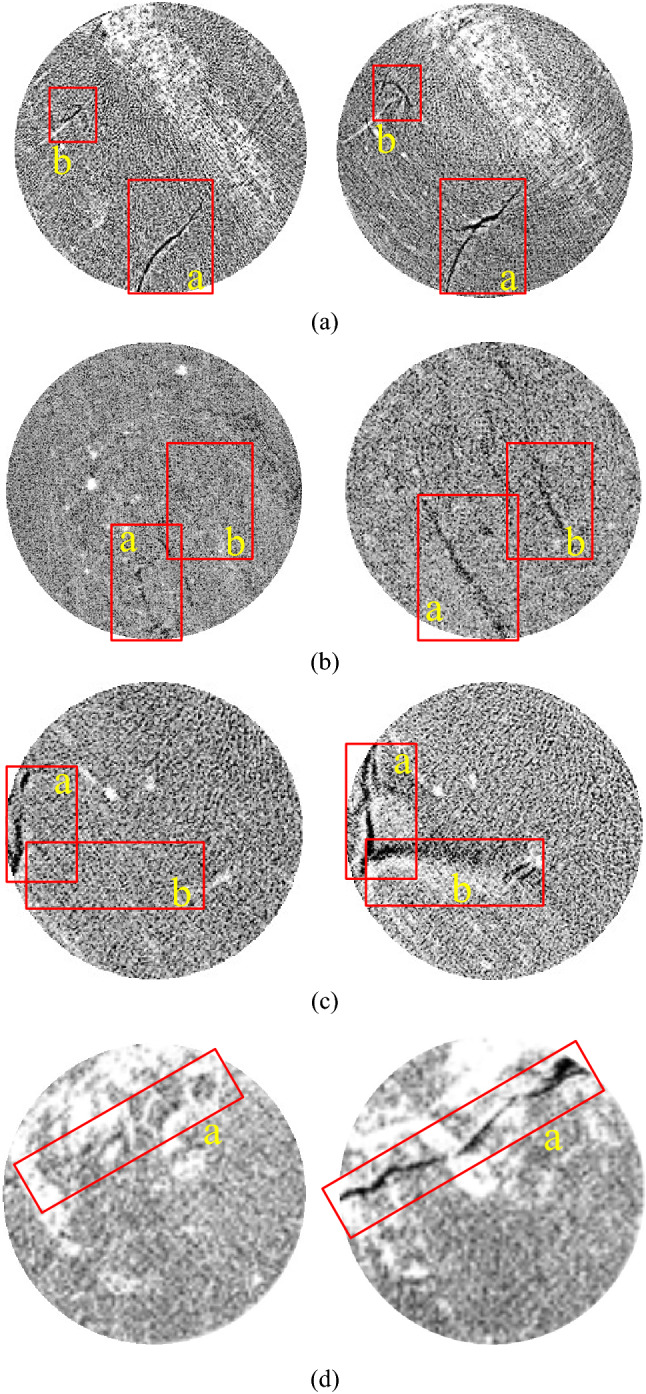
CT observation of coal sections before and after ultrasonic irradiating. (**a**) Sample ^#^4 at No.5 section, (**b**) Sample ^#^5 at No.3 section, (**c**) Sample ^#^6 at No.6 section, (**d**) Sample ^#^10 at No.1 section.

To further verify the fundamental mechanism promoting the permeability and desorption of gassy coal using ultrasonic irradiation, the cleat properties of the samples before and after ultrasonic irradiation were scanned by CT analysis. The CT scans were performed using a 225-kV FCB μCT scanner (Institute of Applied Electronics, CAEP) with a magnification of 1 × to 400 ×. We selected a diameter of 50 mm for each coal sample section, where six layers were divided along the axial direction, as shown in Fig. 1c, and the resolution under the operational conditions stated here was approximately 250 × 250 μm^2^. A list of CT images of the samples is presented in Fig. 12.

For the result presented in Fig. 12, significant changes have occurred in these areas before and after irradiation, as marked with red and named “a” and “b”. With the increase in the area or length of the black, the fractures had expanded under the irradiation of ultrasonic waves, which is the result of the dynamic evolution of cracks from meso to macro caused by the stress concentration formed at the original damage, as confirmed by the Griffith strength criterion and damage mechanics. This damage provides much temporary storage spaces after gas desorption, and more effective penetration channels for gas migration, the gas has a higher flow rate at the same pressure gradient. Moreover, with an increase and communicate of the voids in coal, there is a smaller gas pressure during desorption, and more adsorptive gas is transformed into a free state and migrates into the open areas. In general, the higher sound intensity caused the grate-irradiating force on the sample, and a larger channel formed for gas migration, which is beneficial for gas recovery in coalbeds.

The relationship between the permeability of coal under ultrasound irradiation and gas pressure can be expressed in the form of Eq. (4) combined with Eq. (3), where *a*_1_, *b*_1_, *c*_1_, *a*_2_, *b*_2_, and *c*_2_ are the fitting parameters (mD, mD/MPa, mD/MPa^2^, mD W/cm^−2^, mD W/cm^−2^ MPa^−1^, and mD W/cm^−2^ MPa^−2^); *k*_0_ is the absolute permeability without ultrasound, and the second is the permeability increment caused by the intensity of the ultrasound, both of which are associated with gas pressure. Further simplified, the permeability of coal with ultrasound irradiation can be expressed by Eq. (4). This demonstrates that, in the range of ultrasonic influence, the increase in coal sample permeability is mainly caused by ultrasonic sound intensity and extra increment, the gas permeability rate increases significantly with the increase in pore pressure^40^, and also with a longer irradiation duration with more energy input. As the measurement result in the present paper shows in Figs. 3 and 6, with a higher sound intensity and longer irradiation duration, the permeability of the samples increased, and the corresponding fitting parameters improved. Thus, the ultrasonic waves have a significant effect on improving the permeability in low coal reservoirs.

4 $$ \begin{aligned} k & = e^{{(a_{1} + b_{1} P + c_{1} P^{ \wedge } 2)}} \cdot e^{{(a_{2} J_{0} + b_{2} J_{0} P + c_{2} J_{0} P^{ \wedge } 2)}} = k_{0} e^{{(a_{2} J_{0} + b_{2} J_{0} P + c_{2} J_{0} P^{ \wedge } 2)}} \\ & = e^{{\left[ {\left( {a_{1} + a_{2} J_{0} } \right) + \left( {b_{1} + b_{2} J_{0} } \right)P + \left( {c_{1} + c_{2} J_{0} } \right)P^{ \wedge } 2} \right]}} \\ \end{aligned} $$

In previous studies^41^, the relationship between the gas desorption volume (*Q*_*t*_) and time was similar to the Langmuir adsorption curve, as shown in Eq. (5), where *d* is the saturated desorption and *e* is the parameter (mL and s^−1^). For the desorption of coal under ultrasound irradiation, the saturated desorption can be defined as the product of the proportional coefficient (*A*), mL cm^2^/W, and the effective sound intensity (*J*_0_). Then, the relationship between volume and duration can be expressed in the form of Eq. (7). This demonstrates that, with a higher sound intensity irradiation, the kinetic energy of the gas is increased, and the gas adsorbed internally and on the surface are more easily desorbed and freed. In addition, as the irradiation duration increases, more cracks emerge in the coal for transport, and more gas is collected.

5 $$ Q_{t} = \frac{det}{{1 + et}} $$

6 $$ d = AJ_{0} $$

7 $$ Q_{t} = \frac{{AJ_{0} et}}{1 + et} $$

## Conclusion

In this study, a triaxial stress ultrasonic irradiating test apparatus was developed specifically for coal, considering the properties of gas adsorption, migration, and sound intensity, providing simultaneous gas flux measurements, and enhancing the permeability and desorption for gas recovery from a coal reservoir. The entire deformation and temperature of the coal samples obtained from a depth of 950 m in an underground mine with high gas pressure and content were investigated by permeability and desorption experiments with or without ultrasonic irradiation. The former included three levels of confining pressure, six gas pressure, three irradiation durations and three sound intensities. The variation in the internal structure before and after irradiation was observed using CT scanning. Based on the findings of this study, we draw the following conclusions:

1. With ultrasonic irradiation, the gas permeability of coal is still accompanied by the obvious Klinkenberg effect, as the ultrasonic irradiation duration increases, the permeability of coal improves gradually at each gas pressure with unequal variation. The growth rate of permeability increases with longer irradiation and larger sound intensity but decreases with higher gas and hydrostatic pressure. The variation of temperature was “S shaped” with four stages, with the increase in sound intensity and hydrostatic pressure, which had a higher stable value and greater growth gradient, result in the difference of fracture toughness under hydrostatic stress conditions. Moreover, the strain evolution also developed with a growth rate and final value, which were shown as two phases of micro- and macro- expansion.
2. The desorption of coal is enhanced by the cracks development due to mechanical effect, and thermal effect provided additional energy to increase molecular kinetic energy thermal of ultrasound irradiation, the volume of gas desorption was much greater than that of the sample without mange, and it increased with the sound intensity. The changes in temperature and strain simultaneously displayed a “J shaped” change that increased rapidly following an initial slow increase, which was affected by the gas desorption attracting volumetric shrinkage and temperature reduction.
3. CT scans demonstrated that the fractures had expanded under the irradiation of ultrasonic waves, the resulting dynamic evolution of cracks from meso to macro caused by the stress concentration formed at the original damage, which provides the channel for gas desorption and migration under a pressure gradient. A model of permeability and desorption developed to describe the improvement of coal seam gas production capacity, which introduced effective sound pressure and described the enhancement mechanism of ultrasonic irradiation. Further studies should focus on investigating the influence of the quantified ultrasonic intensity and excitation frequency on the permeability of coal and its engineering applications.

### Ethical statement

I certify that this manuscript is original and has not been published and will not be submitted elsewhere for publication. And the study is not split up into several parts to increase the quantity of submissions and submitted to various journals or to one journal over time. No data have been fabricated or manipulated (including images) to support your conclusions. No data, text, or theories by others are presented as if they were our own. The submission has been received explicitly from all co-authors. And authors whose names appear on the submission have contributed sufficiently to the scientific work and therefore share collective responsibility and accountability for the results.
